# RNA sequencing-based approaches to identifying disulfidptosis-related diagnostic clusters and immune landscapes in osteoporosis

**DOI:** 10.18632/aging.205813

**Published:** 2024-05-10

**Authors:** Peng Zhang, Bing Li, Honglin Chen, Zhilin Ge, Qi Shang, De Liang, Xiang Yu, Hui Ren, Xiaobing Jiang, Jianchao Cui

**Affiliations:** 1 Guangzhou University of Chinese Medicine, Guangzhou 510405, China; 2 The First Affiliated Hospital of Guangxi University of Chinese Medicine, Nanning 530023, China; 3 The First Affiliated Hospital of Guangzhou University of Chinese Medicine, Guangzhou 510405, China; 4 The Second Affiliated Hospital of Guangzhou Medical University, Guangzhou 510260, China

**Keywords:** osteoporosis, disulfidptosis modulator, subtype classification, risk prediction, RNA sequencing

## Abstract

Disulfidptosis, a newly recognized cell death triggered by disulfide stress, has garnered attention for its potential role in osteoporosis (OP) pathogenesis. Although sulfide-related proteins are reported to regulate the balance of bone metabolism in OP, the precise involvement of disulfidptosis regulators remains elusive. Herein, leveraging the GSE56815 dataset, we conducted an analysis to delineate disulfidptosis-associated diagnostic clusters and immune landscapes in OP. Subsequently, vertebral bone tissues obtained from OP patients and controls were subjected to RNA sequencing (RNA-seq) for the validation of key disulfidptosis gene expression. Our analysis unveiled seven significant disulfidptosis regulators, including FLNA, ACTB, PRDX1, SLC7A11, NUBPL, OXSM, and RAC1, distinguishing OP samples from controls. Furthermore, employing a random forest model, we identified four diagnostic disulfidptosis regulators including FLNA, SLC7A11, NUBPL, and RAC1 potentially predictive of OP risk. A nomogram model integrating these four regulators was constructed and validated using the GSE35956 dataset, demonstrating promising utility in clinical decision-making, as affirmed by decision curve analysis. Subsequent consensus clustering analysis stratified OP samples into two different disulfidptosis subgroups (clusters A and B) using significant disulfidptosis regulators, with cluster B exhibiting higher disulfidptosis scores and implicating monocyte immunity, closely linked to osteoclastogenesis. Notably, RNA-seq analysis corroborated the expression patterns of two disulfidptosis modulators, PRDX1 and OXSM, consistent with bioinformatics predictions. Collectively, our study sheds light on disulfidptosis patterns, offering potential markers and immunotherapeutic avenues for future OP management.

## INTRODUCTION

Osteoporosis (OP), as a prevalent skeletal disorder, is featured with loss of bone mass and structural alterations, rendering bones more susceptible to fractures and associated complications, such as pain, deformity, and heightened mortality risk [1–3]. Postmenopausal women, accounting for approximately 50% of individuals, are particularly vulnerable to OP-related fractures [4]. Current therapeutic strategies encompass a spectrum of interventions, including calcium, vitamin D, teriparatide, denosumab, and bisphosphonates [5]; however, prolonged administration of these agents may precipitate adverse reactions, including accelerated bone loss and heightened susceptibility to jaw osteonecrosis, femur fractures, and multiple vertebral fractures experiencing recompression [6]. Consequently, effective clinical management of OP remains elusive [7], posing substantial challenges to healthcare systems and society at large [8]. Given the detrimental impact of OP on health, quality of life, and economic burden, early identification of high-risk individuals assumes paramount importance. Mounting evidence underscores the multifaceted nature of OP, emphasized by its considerable heterogeneity and genetic variability [9]. Accordingly, early risk stratification guided by genetic predispositions holds considerable promise in augmenting OP control efforts.

Disulfidptosis, a recently discovered cell death resulted from disulfide stress, emerges particularly under conditions of glucose deprivation, wherein cells exhibiting heightened SLC7A11 expression and aberrant accumulation of disulfide and cystine components undergo rapid demise due to NADPH consumption-induced disulfide stress [10]. This distinctive mode of cell death implicates a myriad of disulfide-regulatory markers, including FLNA, FLNB, SLC7A11, ACTB, MYH9, SLC3A2, TLN1, OXSM, PRDX1, LRPPRC, NUBPL, RPN1, NCKAP1, NDUFS1, RAC1, WAVE2, NDUFA11, and GYS1 [10]. Prior investigations have underscored the potential of sulfide-regulatory biomarkers as viable targets for diagnosis and therapeutic intervention in OP [11]. Subsequent studies have elucidated the involvement of the sulfide quinone reductase-like gene in modulating osteoblast differentiation [12], while highlighting disulfide’s suppressive effect on osteoclast differentiation via the NF-κB/NFATc1 signalling pathway, thereby mitigating inflammatory osteolysis [13]. Notably, the emergence of disulfidptosis-related ribophorin I (RPN1) is reported to be a therapeutic target and diagnostic biomarker for OP, particularly in the context of kaempferol intervention [14]. These collective findings prompt speculation on the pivotal role of disulfidptosis in the pathological progress of OP through the modulation of disulfide stress-related protein expression. Nevertheless, the precise contributions of disulfidptosis modulators to OP pathogenesis remain elusive.

**Figure 1:**
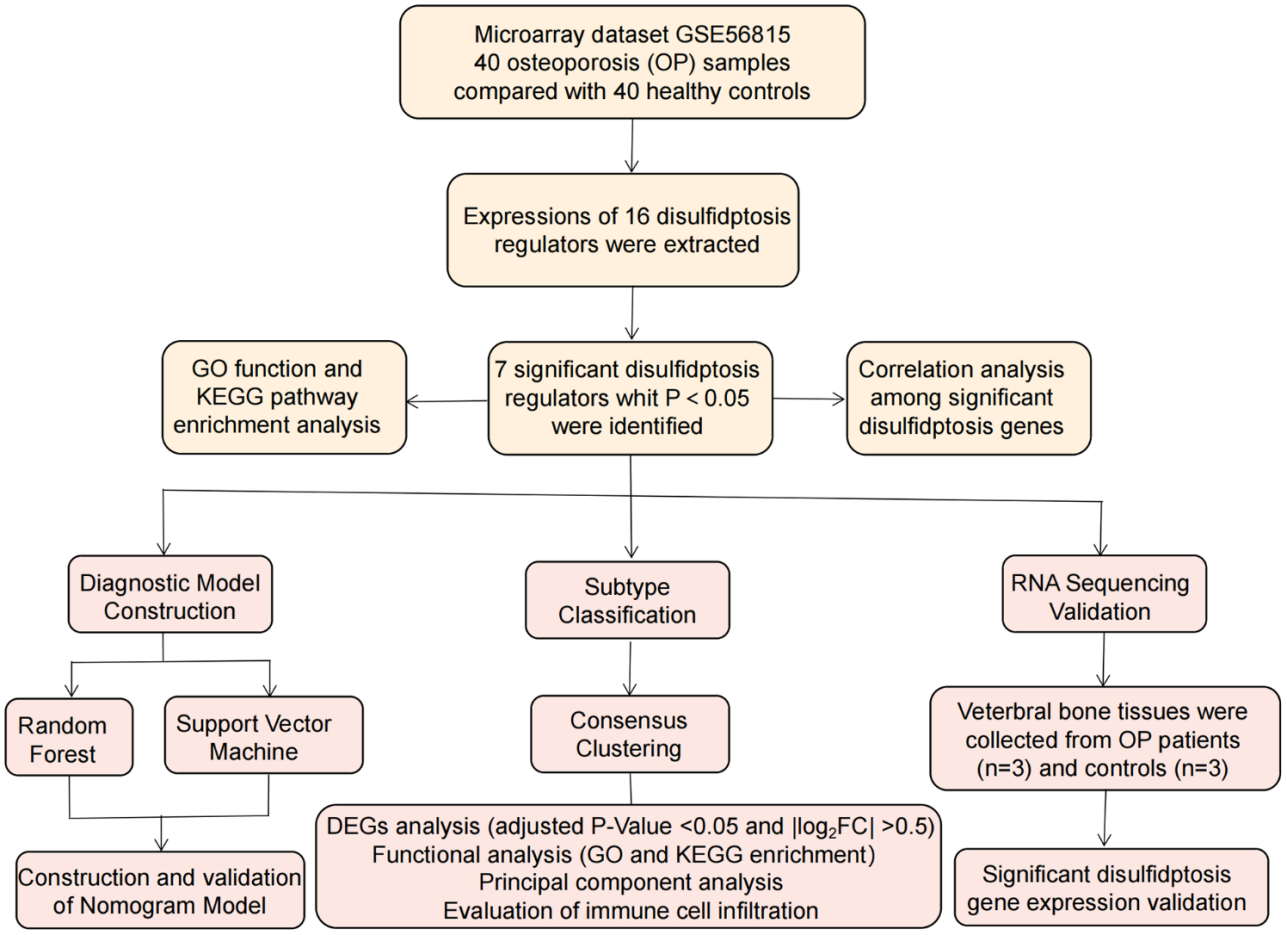
Flowchart of the study design.

Utilizing the GSE56815 dataset [15], we investigated the relevance of disulfidptosis regulators in delineating OP subtypes and identifying potential diagnostic biomarkers. A predictive model for OP susceptibility was devised, integrating seven putative disulfidptosis regulators (FLNA, ACTB, PRDX1, SLC7A11, NUBPL, OXSM, and RAC1), demonstrating utility in clinical practice. Validation of these disulfidptosis regulators was conducted via RNA sequencing (RNA-seq), confirming expression patterns consistent with bioinformatics predictions. Furthermore, our analysis unveiled two distinct disulfidptosis patterns, exhibiting significant associations with many immune cells like monocytes. These findings underscore the potential of disulfidptosis patterns as diagnostic markers for OP, offering insights into tailored immunotherapeutic interventions. Methodological approaches employed in this study align with previous published literature [16–18]. The flow chart of research process is described in Figure 1.

## MATERIALS AND METHODS

### Sample data retrieval for OP analysis

Utilizing the GEO data repository (http://www.ncbi.nlm.nih.gov/geo/), we accessed data pertaining to monocytes extracted from whole blood specimens of OP patients. The search parameters encompassed terms such as ‘osteoporosis’, ‘BMD’, ‘gene expression’, and ‘microarray’. Selection criteria included datasets comprising a minimum of 60 samples, with at least 30 cases each in the control and OP cohorts, alongside availability of downloadable series matrix files and raw data. Ultimately, we opted for the GSE56815 dataset [15]. Within this dataset, we have pinpointed 40 samples each in the OP and control groups deemed eligible for further analysis.

### Data collection

The conversion of microarray probes into symbols was achieved in the R software (v4.1.2), utilizing the annotation package obtained from the Bioconductor website(http://bioconductor.org/). Subsequently, the dataset underwent quantile normalization to ensure uniformity across samples, comprising 40 control and 40 OP cases. Utilizing the Limma R package, distinct disulfidptosis regulators within the dataset were identified through comparative analysis between control and OP cohorts, employing significance cutoff of |log_2_ fold change (FC)| > 0 and *P* < 0.05 [19]. Furthermore, the clusterProfiler R package facilitated the enrichment analyses of GO and KEGG to investigate potential mechanisms of action associated with disulfidptosis regulators in OP.

### Model establishment

In our investigation of OP occurrence, we devised two models of support vector machine (SVM) and random forest (RF) as testing frameworks, employing various methodologies including ROC curve, reverse cumulative residual distribution, and residual box plots [17]. The RandomForest package for R environments was utilized to construct the RF model, selecting putative disulfidptosis mediators based on significance scores (Mean Decrease Gini) [16]. For the SVM model, the parameter *n* denoted disulfidptosis gene number, with every data point depicted as a singular position situated within an *n*-dimensional realm. Subsequently, an optimal hyperplane was identified to effectively segregate the OP and control groups [20]. Furthermore, we devised a nomogram signature utilizing the R plugin rms, facilitating OP prediction based on selected candidate disulfidptosis regulators. To validate our model, we employed the GSE35956 dataset [21], comprising 5 OP samples and 5 controls subjected to analysis. Calibration curves were adopted to gauge the alignment between prediction values and actual outcomes. Additionally, clinical impact curve was constructed through decision curve analysis (DCA), assessing the utility of model-based decisions in patient management [16].

### Subtype classification

Utilizing consensus clustering with resampling, every element and its corresponding subgroup number were determined with validation confirming the cluster coherence [16]. The ConsensusClusterPlus package for R environments was employed to unveil different disulfidptosis subtypes according to significant disulfidptosis regulators [22].

### GO enrichment analysis of DEGs

Employing the Limma package for R environments, we discerned differentially expressed genes (DEGs) between disulfidptosis clusters adopting adjusted *P* < 0.05 and |log_2_ FC| > 0.5 as thresholds. Subsequent GO enrichment analysis, performed by the R plugin clusterProfiler, elucidated the potential involvement of DEGs in OP pathogenesis [23].

### Disulfidptosis score assessment

Disulfidptosis scores for individual samples were computed through principal component analysis (PCA) quantifying disulfidptosis patterns. The evaluative formula utilized for calculating the disulfidptosis score was disulfidptosis score = PC1_i_, where PC1 represents principal component 1 and i denotes crucial disulfidptosis gene expression [24].

### Assessment of immune cell abundance

Immune cell infiltration in OP cases was evaluated via single-sample gene set enrichment analysis (ssGSEA). Initially, ssGSEA determined gene expression levels in samples, generating a ranking based on expression. Subsequently, the expression levels of key regulators of disulfidptosis were assessed, and their cumulative expression served to quantify immune cell populations within each sample [25].

### RNA-seq analysis of vertebral bone samples from the OP and control groups to verify differential expression of disulfidptosis genes

Vertebral bone tissue samples were collected from individuals diagnosed with osteoporosis (OP) and control participants undergoing surgical treatment First Affiliated Hospital of Guangzhou University of Chinese Medicine. RNA-seq analysis was then employed to explore differential expressions of disulfidptosis-related genes between the two groups. Notably, no significant difference was noted in age, body mass index, serum phosphorus, serum alkaline phosphatase, and serum magnesium levels between the two groups. However, serum calcium levels, lumbar spine (L1–L4) T-score, and bone mineral density were significantly lower in the OP group in comparison to the control group (*P* < 0.05). Total RNA samples were collected from three OP patients and three control participants utilizing Trizol reagent. These RNA samples underwent agarose gel electrophoresis, Nanodrop quality inspection and quantification. The mRNA enrichment was performed using oligo magnetic beads, followed by library establishment for Illumina sequencing (Kapa Biosystems, Woburn, MA, USA). Library quality was evaluated, and quantification was conducted by quantitative polymerase chain reaction.

Subsequently, libraries from different samples were pooled based on quantitative results and final data for sequencing. DEGs in the dataset were screened by analyzing differences between control and OP samples adopting the Limma R package. Then, disulfidptosis modulators were identified and their expression profiles were constructed from the data. The screening thresholds used to detect disulfidptosis DEGs were set at |log_2_FC| > 1 and *P* < 0.05.

### Statistical analysis

The analysis of linear regression was utilized to assess the relationships between crucial disulfidptosis regulators. For bioinformatics analysis, group-wise comparisons were conducted using the Kruskal–Wallis test, while corrected *t*-tests were employed to analyze RNA-seq data. The parametric analysis was conducted using two-tailed tests, with statistical threshold set at *P* < 0.05.

## RESULTS

### Screening of 16 disulfidptosis regulators in OP

**Figure 2:**
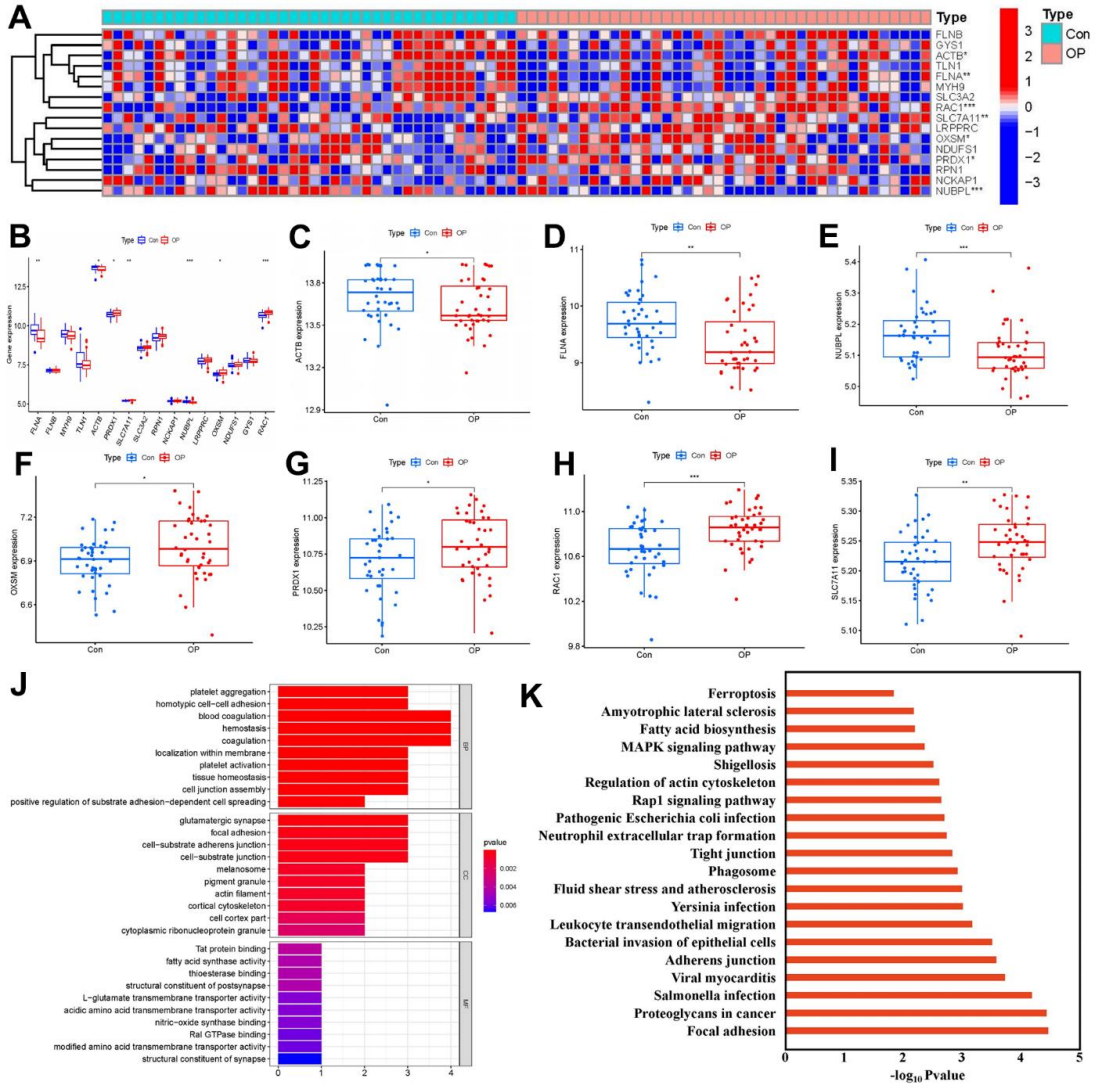
**Identification of 16 disulfidptosis modulators in OP.** (**A**) Expression heat map of 16 disulfidptosis modulators in OP cases and controls. (**B**) Differential expression box plot of 16 disulfidptosis modulators identified between OP cases and controls. (**C**–**I**) Differential expression box plots of seven significant disulfidptosis modulators identified between OP cases and controls. (**J**, **K**) GO and KEGG enrichment analysis based on seven significant disulfidptosis modulators. *P < 0.05, **P < 0.01, ***P < 0.001.

In total, 16 regulators associated with disulfidptosis were identified by analyzing gene expression profiles from both control and OP cases. From this analysis, seven distinct regulators (FLNA, ACTB, PRDX1, SLC7A11, NUBPL, OXSM, and RAC1) emerged, depicted in a heat map and box plot (Figure 2A, 2B). Notably, ACTB, FLNA, and NUBPL showed reduced expression levels, while PRDX1, SLC7A11, OXSM, and RAC1 exhibited increased expression in OP cases compared to controls (Figure 2C–2I). Additionally, GO analysis highlighted significant enrichment in biological process (tissue homeostasis), cellular components (glutamatergic synapses and actin filaments), and molecular function (L-glutamate transmembrane transporter activity) (Figure 2J). Furthermore, regulation of actin cytoskeleton and ferroptosis pathways emerged as the main enriched pathways according to KEGG analysis (Figure 2K). Detailed information regarding the GO and KEGG enrichment analyses can be found in Supplementary Tables 1, 2.

### Correlations among disulfidptosis regulators in OP

**Figure 3:**
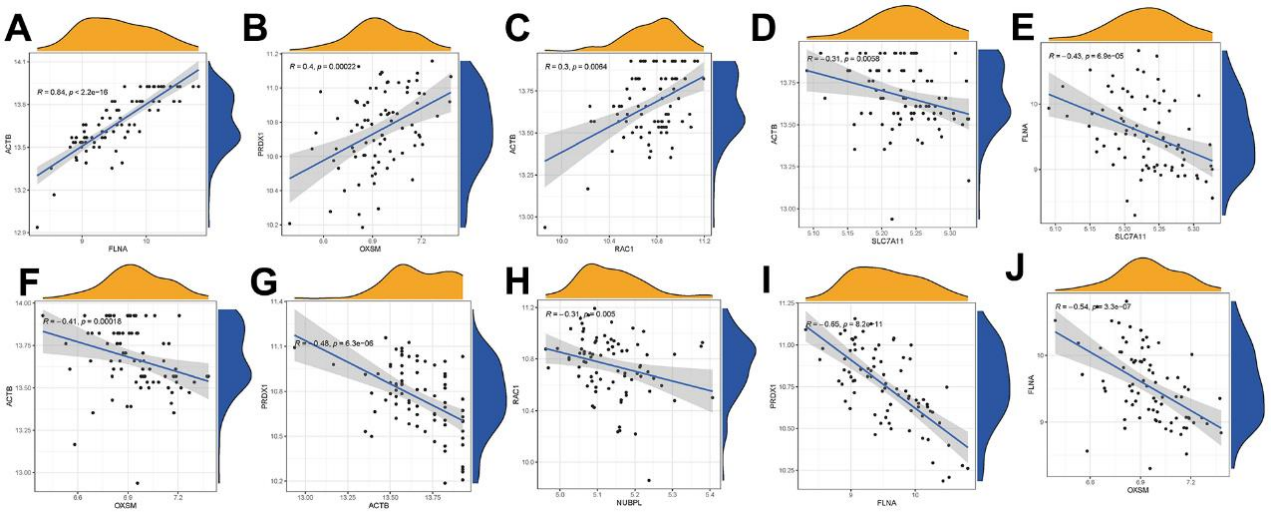
Correlations among disulfidptosis modulators in OP (**A**–**J**). In OP cases, there were significantly positive correlations in gene expression levels of ACTB–FLNA, ACTB–RAC1, and PRDX1–OXSM (**A**–**C**); there were significantly negative correlations in gene expression levels of ACTB–SLC7A11, FLNA–SLC7A11, ACTB–OXSM, PRDX1–ACTB, RAC1–NUBPL, PRDX1–FLNA, and FLNA–OXSM (**D**–**J**).

In OP samples, there were significantly positive associations observed in the gene expressions of ACTB–FLNA, ACTB–RAC1, and PRDX1–OXSM (Figure 3A–3C). Conversely, significantly negative correlations were noted in the gene expressions of ACTB–SLC7A11, FLNA–SLC7A11, ACTB–OXSM, PRDX1–ACTB, RAC1–NUBPL, PRDX1–FLNA, and FLNA–OXSM (Figure 3D–3J). These observations underscore distinct correlations among various disulfidptosis modulators in OP.

### Establishment of RF and SVM models

**Figure 4:**
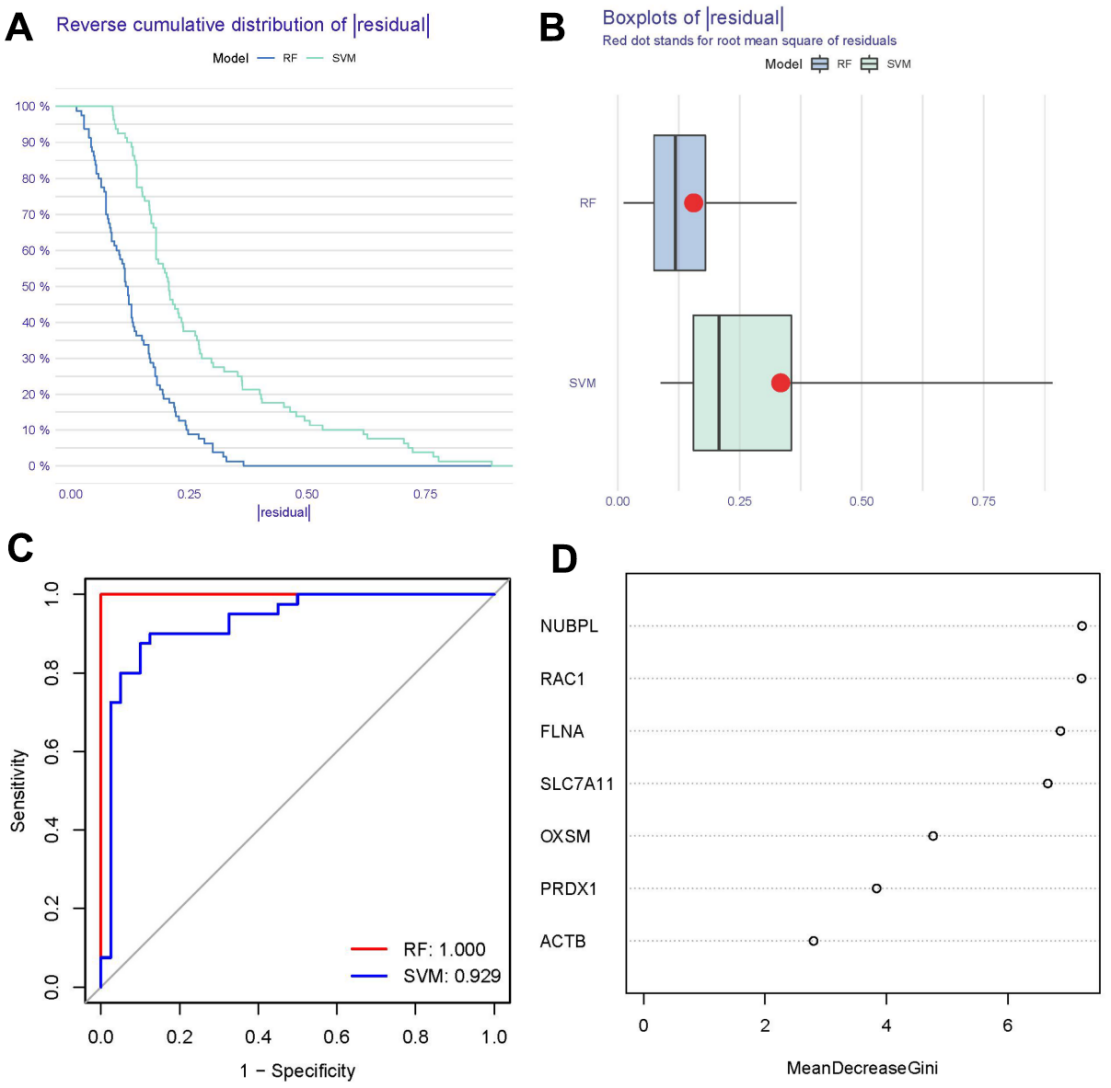
**Establishment of RF and SVM models.** (**A**) Reverse cumulative distribution constructed to display the residual distributions of RF and SVM models. (**B**) Box plot constructed to display the residual distributions of RF and SVM models. (**C**) ROC curves show the accuracies of the RF and SVM models. (**D**) Importance scores of seven disulfidptosis modulators based on the RF model.

As depicted in Figure 4A, 4B, the RF model exhibited the smaller residuals, indicating its superior performance over the SVM model. Therefore, we utilized the RF model to forecast the occurrence of OP. ROC curve analysis revealed that the RF model outperformed the SVM model in terms of accuracy (Figure 4C). Consequently, we established an RF model to screen candidate disulfidptosis mediators for establishing a nomogram model [16]. Subsequently, the seven significant disulfidptosis regulators were ranked based on importance scores (mean decrease Gini), and four regulators with scores > 6 (FLNA, SLC7A11, NUBPL, and RAC1) were selected as candidates (Figure 4D).

### Nomogram model construction

**Figure 5:**
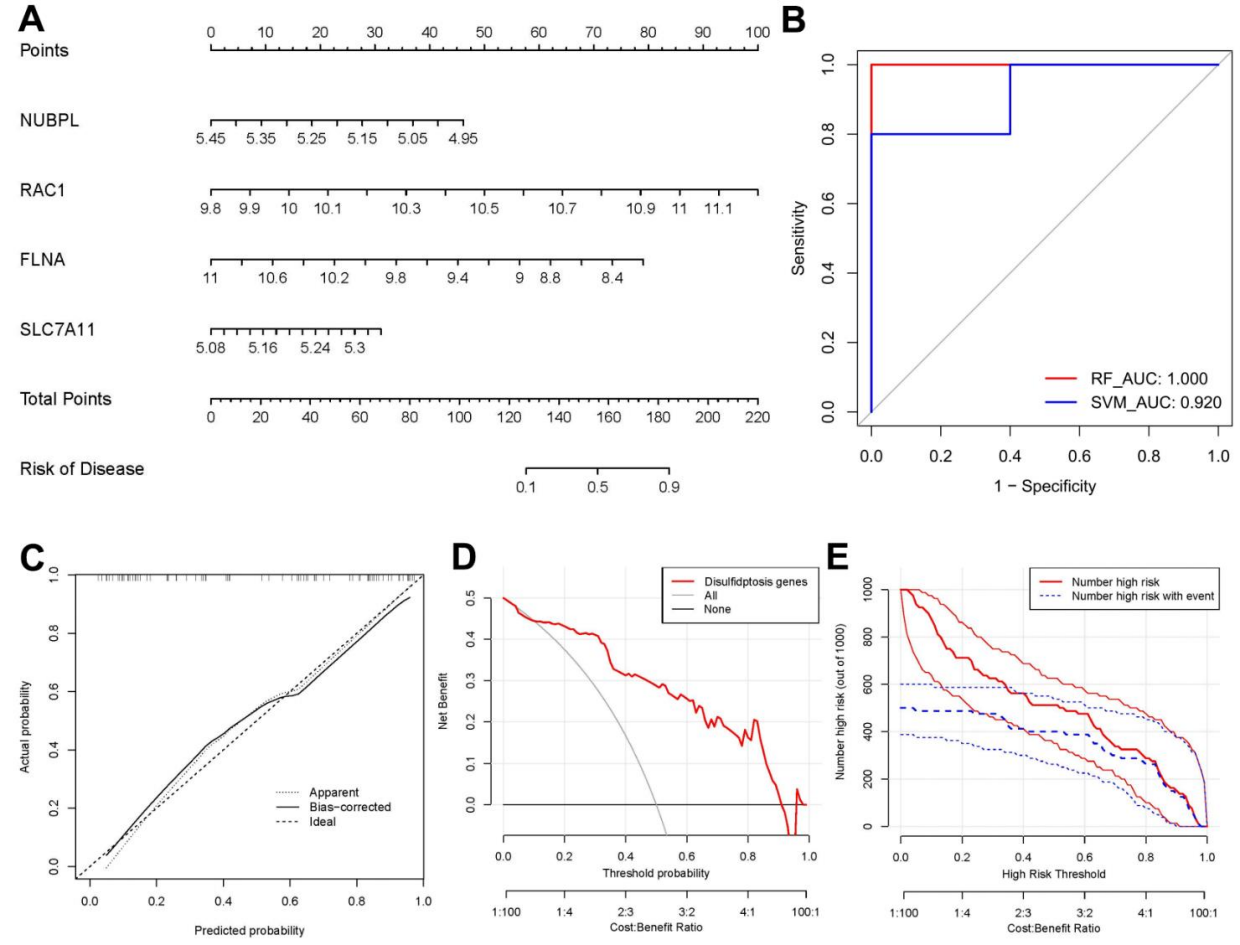
**Establishment of the nomogram model.** (**A**) A nomogram model was established based on four candidate disulfidptosis modulators. (**B**) The ROC result of our proposed signature in validation dataset GSE35956. (**C**) A calibration curve was utilised to evaluate the predictive accuracy of the nomogram model. (**D**) Decisions based on this nomogram model may be beneficial for clinical management of patients with OP. (**E**) A clinical impact curve was used to assess the clinical impact of the nomogram model.

We established a nomogram model utilizing the four nominated disulfidptosis regulators with the R plugin (rms) to forecast OP outcomes (Figure 5A). To validate this model, we utilized the GSE35956 dataset for further verification. The ROC analysis demonstrated notable AUC values, indicating a high level of diagnostic accuracy for this signature (Figure 5B). Additionally, the nomogram model exhibited favourable prediction accuracy as evidenced by calibration curves (Figure 5C). Notably, the DCA plot revealed that the red line continuously surpassed the black and gray lines across the entire probability range from 0 to 1, suggesting potential benefits for OP patients through decisions informed by the nomogram model (Figure 5D). Furthermore, the nomogram model displayed a reliable ability to predict outcomes, as demonstrated by Figure 5E.

### Identification of two different disulfidptosis clusters

**Figure 6:**
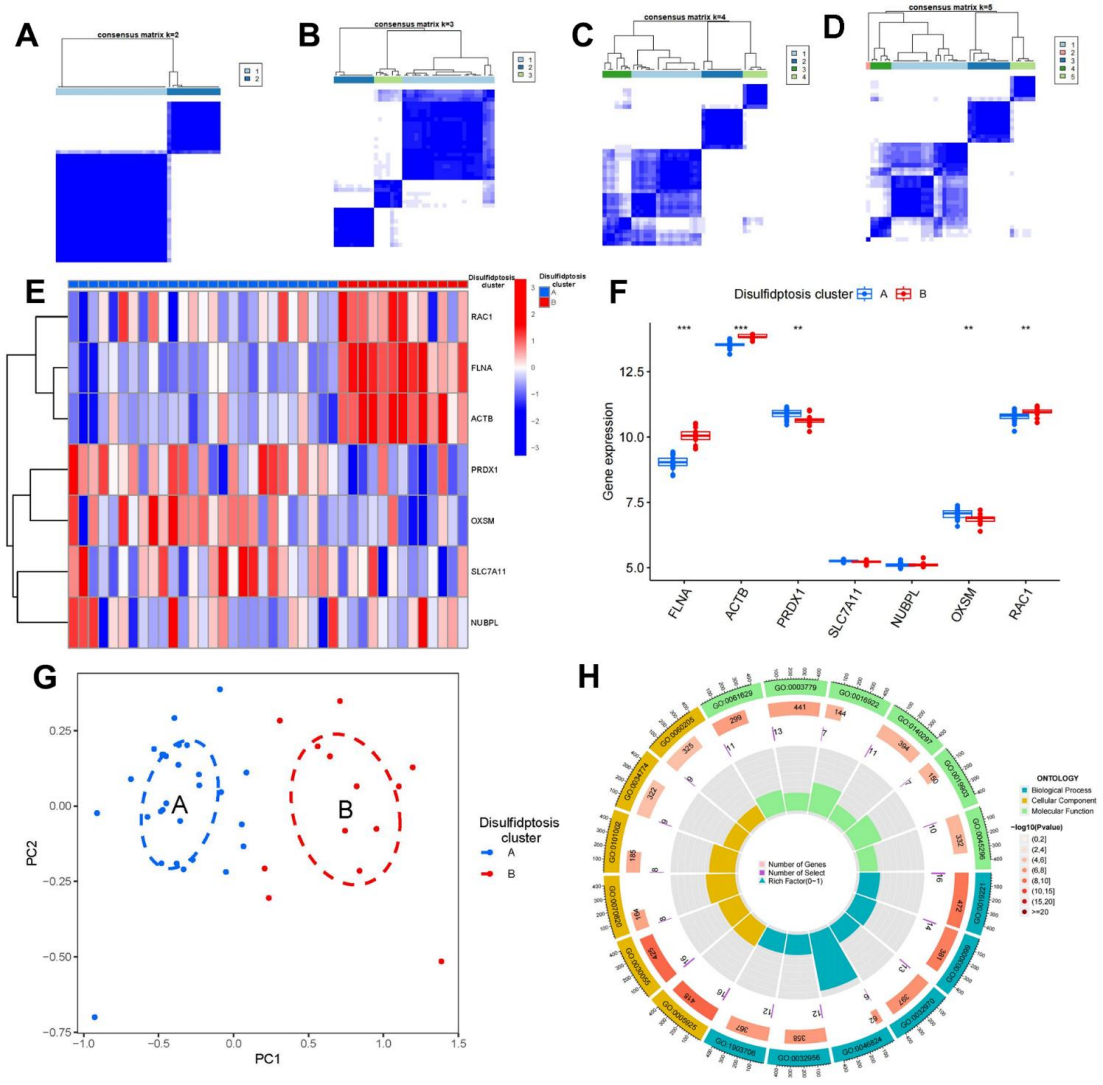
**Consensus clustering of seven significant disulfidptosis modulators in OP.** (**A**–**D**) Consensus matrices of seven significant disulfidptosis modulators for k = 2–5. (**E**) Expression heat map of seven significant disulfidptosis modulators in clusters A and B. (**F**) Differential expression box plots of seven significant disulfidptosis modulators in clusters A and B. (**G**) Principal component analysis of the expression profiles of seven significant disulfidptosis modulators showing substantial differences in transcriptomes between the two disulfidptosis patterns. (**H**) GO enrichment analysis was performed to explore potential mechanisms underlying the effects of 127 disulfidptosis-related DEGs on the occurrence and development of OP. **P < 0.01, ***P < 0.001.

Through the utilization of ConsensusClusterPlus, we identified two distinct disulfidptosis clusters, denoted as clusters A and B, according to the expression profiles of the seven disulfidptosis regulators (Figure 6A–6D). Notably, cluster A encompassed 27 samples, while cluster B comprised 13 samples. Differential analysis of the gene expressions in the seven disulfidptosis modulators between these clusters revealed clear distinctions as depicted on heat maps and box plots. Specifically, cluster B demonstrated higher expression levels of FLNA, ACTB, and RAC1, whereas cluster A exhibited elevated expression levels of PRDX1 and OXSM. However, no significant differences were observed in the gene expressions of SLC7A11 and NUBPL between the two clusters (Figure 6E, 6F). PCA corroborated the discriminatory power of the seven disulfidptosis regulators in distinguishing between the two disulfidptosis clusters (Figure 6G). Subsequently, we identified 127 DEGs associated with disulfidptosis between the two clusters. To elucidate the functional roles of these DEGs in OP, we conducted GO enrichment analysis (Figure 6H), revealing enrichment terms such as regulation of actin cytoskeleton organization (GO: 0032956), regulation of actin filament-based process (GO: 0032970), cell-substrate junction (GO: 0030055), and actin binding (GO:0003779). The information containing the GO enrichment results was available in Supplementary Table 3.

Subsequently, we proceeded to investigate the associations between the seven pivotal disulfidptosis modulators and immune cells by utilizing ssGSEA to measure the abundance of immune cells in OP samples.

**Figure 7:**
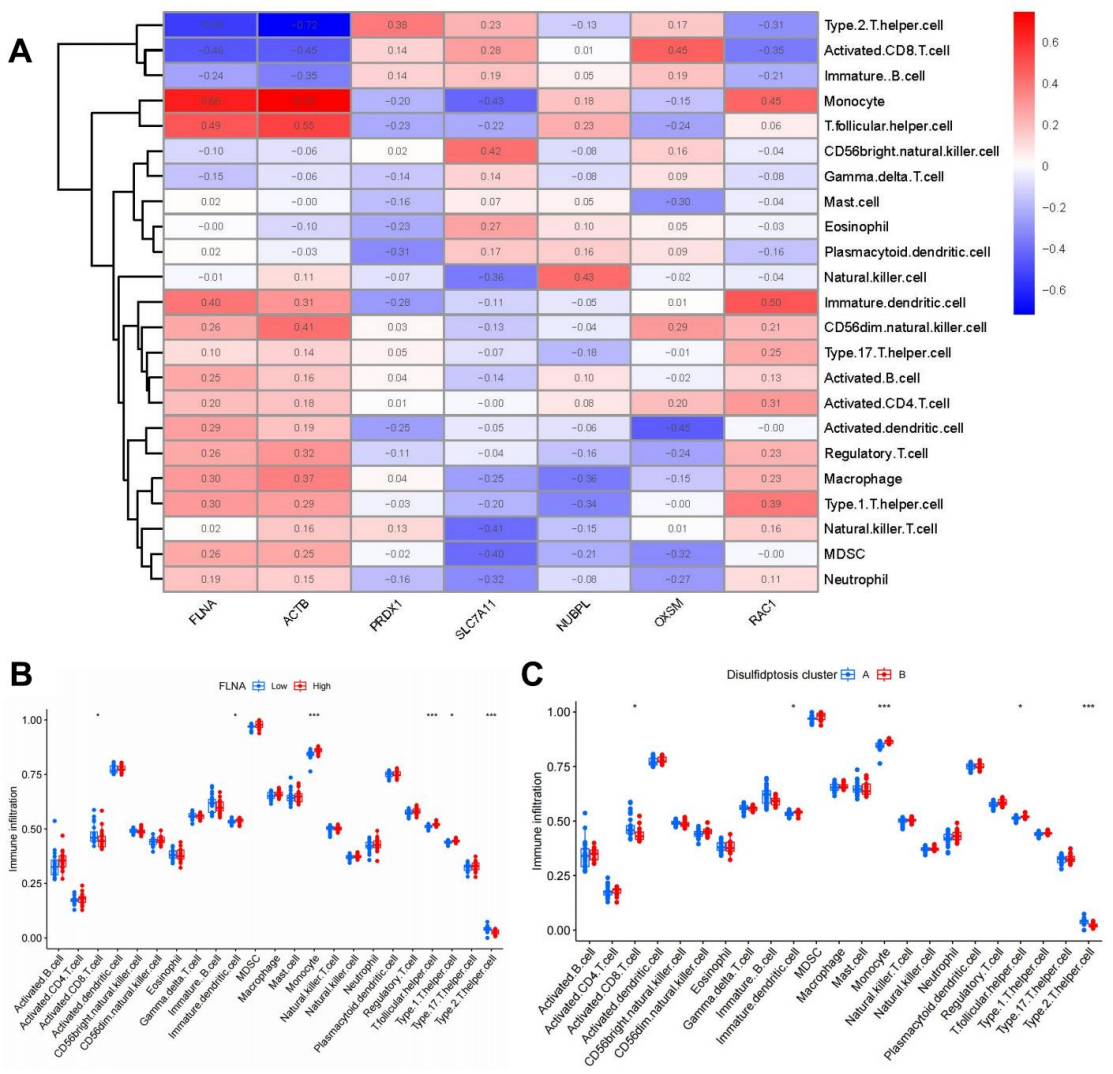
**Single-sample gene set enrichment analysis.** (**A**) Correlations between immune cell infiltration and seven significant disulfidptosis modulators. (**B**) Differences in abundance of infiltrating immune cells between high- and low-FLNA protein expression groups. (**C**) Differential immune cell infiltration between cluster A and cluster B. *P < 0.05, ***P < 0.001.

Positive associations were noted among FLNA expression and several immune cell subsets (Figure 7A). Next, differences in immune cell infiltration were further explored between patients with high and low expression levels of FLNA. Remarkably, individuals with high FLNA expression displayed heightened immune cell infiltration in comparison to those with low FLNA expression (Figure 7B). Cluster B emerged to be more strongly correlated with OP, given its association with immature dendritic cells, monocytes, and T follicular helper cells, which are closely involved in osteoclast differentiation. Conversely, cluster A was associated with activated CD8^+^ T cells and type 2 T helper cells (Figure 7C).

### Disulfidptosis gene cluster construction

**Figure 8:**
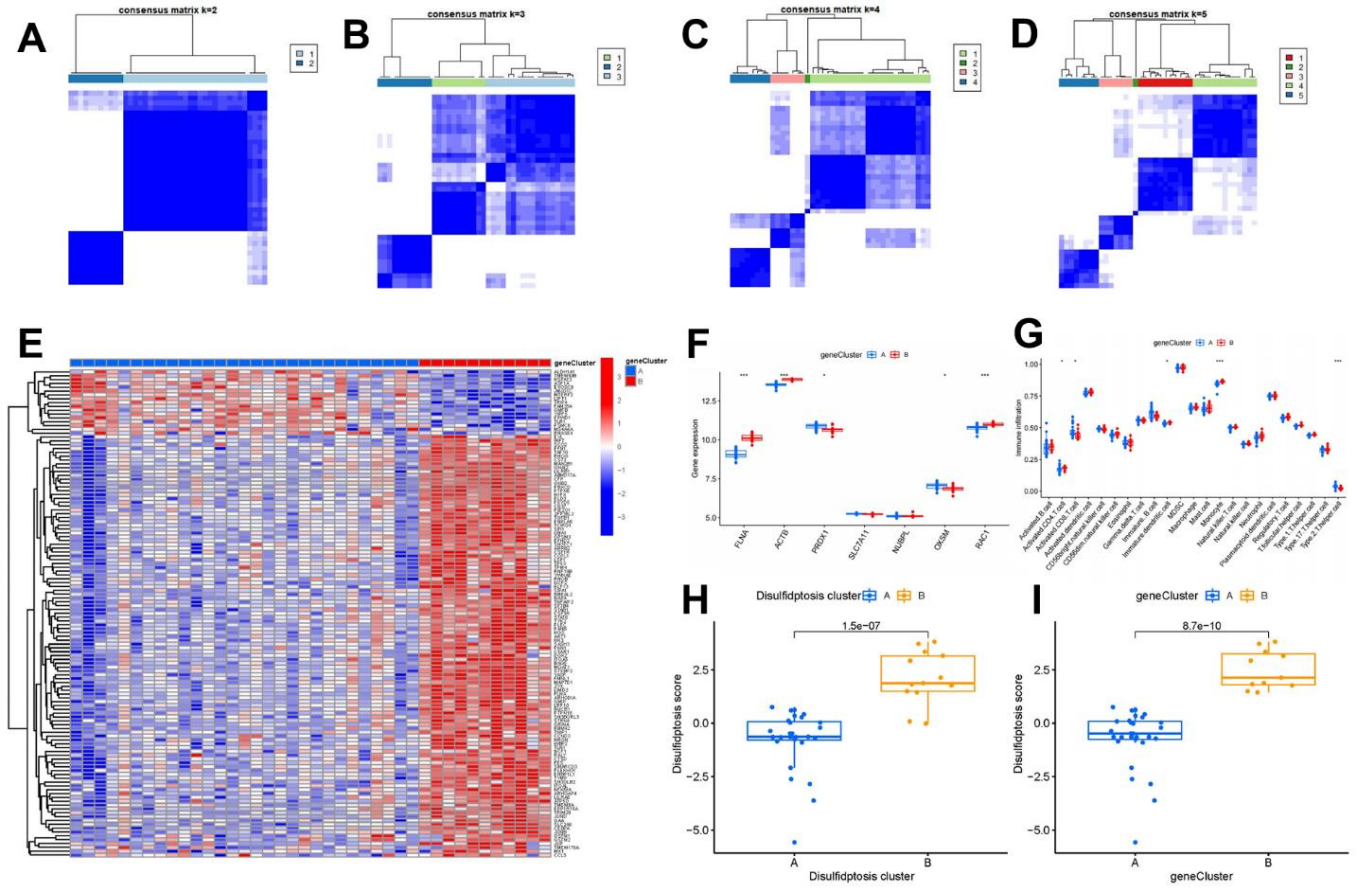
**Consensus clustering of 127 disulfidptosis-associated DEGs in OP.** (**A**–**D**) Consensus matrices of 127 disulfidptosis-associated DEGs for k = 2–5. (**E**) Expression heat map of 127 disulfidptosis-associated DEGs in gene clusters A and B. (**F**) Differential expression box plots of seven significant disulfidptosis modulators in gene clusters A and B. (**G**) Differential immune cell infiltration between gene clusters A and B. (**H**) Differences in disulfidptosis scores between cluster A and cluster B. (**I**) Differences in disulfidptosis scores between gene clusters A and B. *P < 0.05, ***P < 0.001.

To delineate disulfidptosis patterns, we employed a consensus clustering technique to categorize OP samples into distinct genetic subtypes according to 127 disulfidptosis-associated DEGs. The analysis unveiled two distinct disulfidptosis gene clusters, denoted as gene clusters A and B, which aligned with the disulfidptosis clusters previously identified (Figure 8A–8D). Expression profiles of the 127 disulfidptosis-related DEGs in gene clusters A and B are described in Figure 8E. Furthermore, the levels of immune cell infiltration and the gene expressions of the seven vital disulfidptosis regulators in gene clusters A and B were also similar to the disulfidptosis patterns (Figure 8F, 8G), validating the precision of the consensus clustering method. Notably, cluster B and gene cluster B showed higher disulfidptosis scores in comparison to cluster A and gene cluster A (Figure 8H, 8I).

### Role of disulfidptosis clusters in OP identification

**Figure 9:**
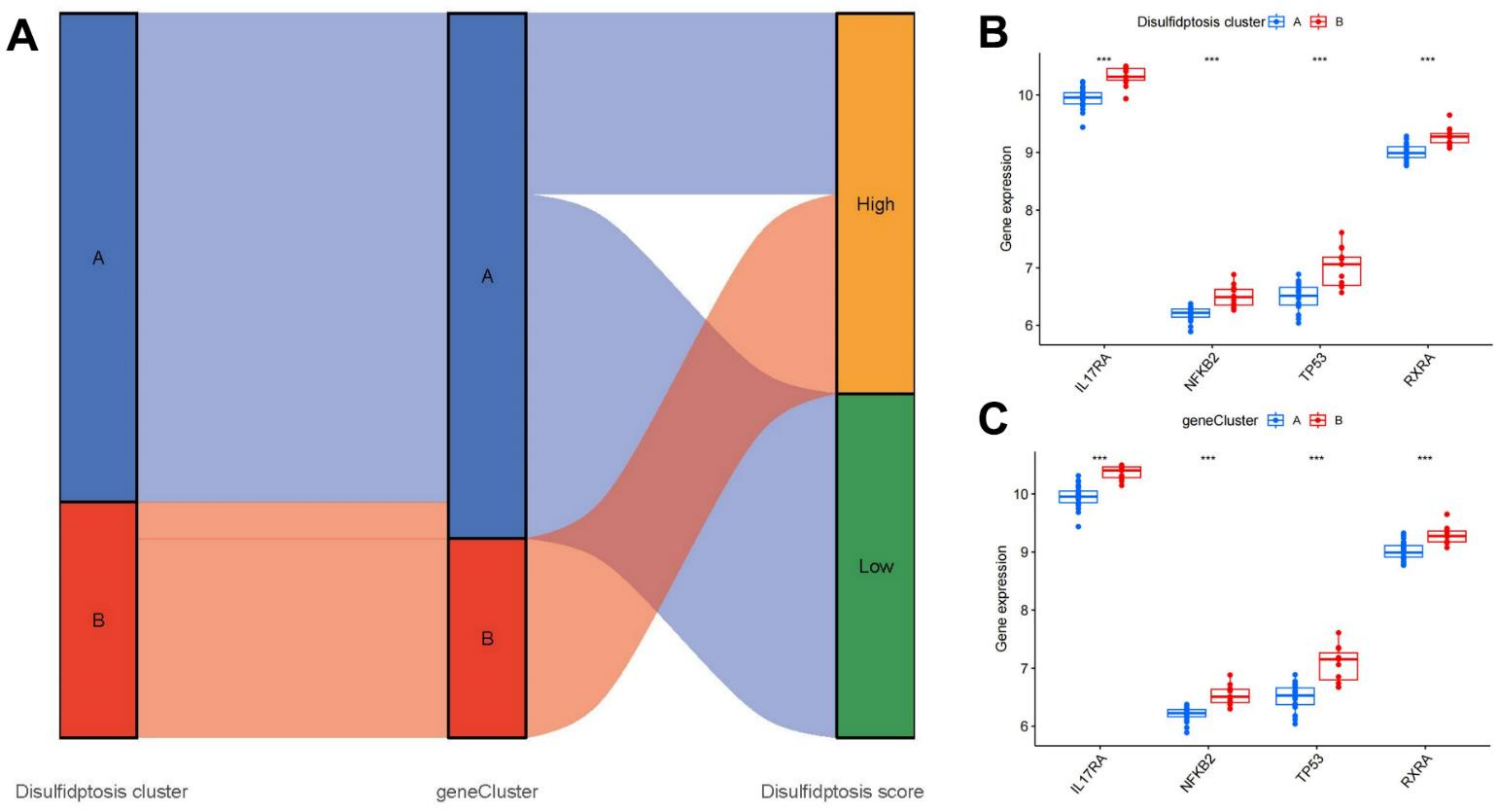
**Roles of disulfidptosis patterns in distinguishing OP.** (**A**) Sankey diagram showing relationships among disulfidptosis patterns, disulfidptosis gene patterns, and disulfidptosis scores. (**B**) Differential expression levels of osteoclast differentiation-related genes between cluster A and cluster B. (**C**) Differential expression levels of osteoclast differentiation-related genes between gene cluster A and gene cluster B. ***P < 0.001.

The associations among disulfidptosis patterns, disulfidptosis scores, and disulfidptosis gene patterns are presented in Figure 9A. To explore the associations between OP and disulfidptosis patterns, we investigated correlations among disulfidptosis patterns and the expression levels of IL17RA, NFKB2, TP53, and RXRA, which are closely linked to osteoclast differentiation. Notably, higher expression levels of IL17RA, NFKB2, TP53, and RXRA were noted in cluster B and gene cluster B compared to cluster A and gene cluster A, suggesting a strong association of cluster B and gene cluster B with OP characterized by osteoclast differentiation (Figure 9B, 9C).

### RNA-seq-based verification of important disulfidptosis regulators

**Figure 10:**
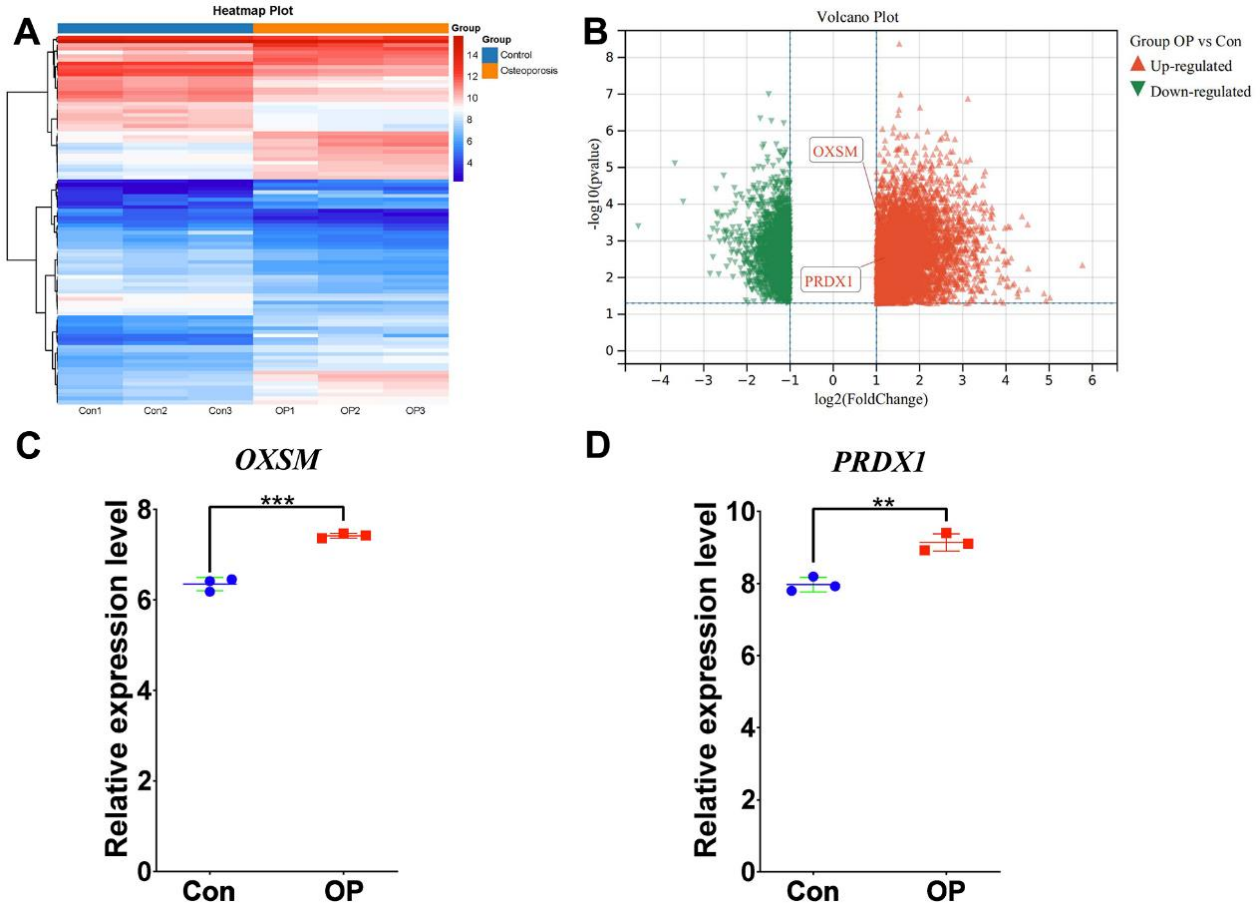
**RNA-seq validation of significant disulfidptosis modulators.** (**A**) Expression heat map and (**B**) volcano plot of vertebral bone tissues from patients with OP and controls, assessed by RNA-seq. (**C**) The disulfidptosis modulator OXSM exhibited increased expression levels in OP samples compared with controls. (**D**) The disulfidptosis modulator PRDX1 exhibited increased expression levels in OP samples compared with controls. All results are expressed as means ± standard deviations. **P < 0.01, ***P < 0.001.

After meticulous filtering, we identified two essential disulfidptosis modulators, PRDX1 and OXSM, which were illustrated via an expression heat map and volcano plot (Figure 10A, 10B). Notably, the disulfidptosis genes OXSM and PRDX1 exhibited significantly elevated expression levels in OP patients compared to controls (Figure 10C, 10D), validating the predictions generated from bioinformatics analyses.

## DISCUSSION

OP is a prevalent musculoskeletal disorder marked by bone-related symptoms [26]. Previous studies have demonstrated that sulfide-related proteins can regulate the balance of bone metabolism in OP, indicating a potential involvement of disulfidptosis in the pathological process of OP [11–13]. However, it remains unclear whether disulfidptosis regulators play crucial roles in OP. The present study explored the roles of disulfidptosis regulators in OP pathology.

In this study, we utilized RF and SVM models to create a gene signature associated with disulfidptosis, employing the seven significant disulfidptosis regulators. The RF model operates as a learning algorithm by assembling independent decision trees derived from random samples. Each decision tree undergoes independent learning and prediction, with the final result being the average of all trees [27, 28]. On the other hand, the SVM model acts as a discriminative classifier, trained on labelled samples to classify test samples using the output of an optimal hyperplane [29]. In comparison to the SVM model, we observed that the RF model had higher AUC value and smaller residual, making it the most suitable testing model. Subsequently, the established RF model predicted the occurrence of OP, highlighting seven significant disulfidptosis regulators with differential expression between OP and control samples (namely, SLC7A11, FLNA, ACTB, PRDX1, NUBPL, OXSM, and RAC1) as potential diagnostic markers. Furthermore, we developed a nomogram model consisting of four potential disulfidptosis regulators (SLC7A11, FLNA, NUBPL, and RAC1) with significance scores > 6, based on DCA analysis of clinical benefits for OP patients.

Under circumstances of glucose deprivation, SLC7A11, also recognized as xCT, facilitates cystine intake and fosters disulfidptosis [10]. The upregulation of SLC7A11 *in vitro* has been demonstrated to significantly impede osteoblast differentiation of mesenchymal stem cells and dampen bone formation *in vivo* [30]. Additionally, SLC7A11 serves as an epigenetic cofactor that disrupts osteoclastogenesis [31]. OP is characterized by excessive osteoclast activity, and hindering osteoclast differentiation effectively shields against OP [32]. Previous study has revealed that NFATc1-induced upregulation of SLC7A11 triggers targetable sensitivity to thioredoxin reductase 1 (TXNRD1) inhibitors during osteoclastogenesis, potentially leading to the selective elimination of osteoclast precursors through intracellular cystine accumulation and subsequent disulfidptosis [33]. As a small GTPase, RAC1 (Ras-related C3 botulinum toxin substrate 1) is upregulated to activate disulfidptosis [10]. During osteoclast differentiation, RAC1 undergoes significant upregulation; its knockdown suppresses osteoclast differentiation and monocyte apoptosis [34]. PRDX1 (peroxiredoxin 1) is a known modulator of reactive oxygen species that regulates oxidative stress and BMP signalling-driven osteogenesis [35]. OXSM (mitochondrial 3-oxoacyl-ACP synthase) reportedly targets miR338-3p [36], which has important functions in regulating bone homeostasis in OP [37]. The present study confirmed that these genes—SLC7A11, RAC1, PRDX1 and OXSM—encoding disulfidptosis activators exhibited higher expression levels in OP samples, thereby activating disulfidptosis processes in OP.

Actin cytoskeleton proteins exhibit high susceptibility to disulfide stress; abnormal disulfide bonding between actin cytoskeleton proteins has the potential to induce the collapse of the actin network and initiate disulfidptosis [10]. The actin cytoskeleton proteins, FLNA (filamin-A) and ACTB (actin), possess numerous cysteine sites that form disulfide bonds under conditions of glucose starvation; Alongside the NUBPL (nucleotide binding protein-like) factor associated with mitochondrial oxidative phosphorylation, these proteins act as disulfidptosis modulators, synergizing with glucose starvation to trigger disulfidptosis [10]. Previous research has shown that inactivation of ACTB blocks osteogenic differentiation and proliferation [38]. Evidence suggests that FLNA is involved in negatively regulating osteogenesis and positively modulating osteoclastogenesis [39]. Loss of NUBPL function triggers mitochondrial dysfunction [40], which could disrupt both bone formation and resorption [41]. Importantly, the present study confirmed that the disulfidptosis regulators ACTB, FLNA, and NUBPL exhibited lower expression levels in OP samples, thus influencing disulfidptosis processes in OP. In general, the seven potential disulfidptosis regulators screened in this study may play crucial roles in OP onset and progression.

There is mounting evidence indicating the significance of monocyte osteoclastogenesis in the pathogenesis of OP [42]. Our study revealed that cluster A was associated with activated CD8^+^ T cell and type 2 T helper cell immunity, while cluster B was related to immature dendritic cell, monocyte, and T follicular helper cell immunity, which are closely associated with osteoclastogenesis; these findings suggest that cluster B is more closely associated with OP (Figure 7C). Monocytes play a pivotal role in maintaining immunological homeostasis and preventing the onset of OP [43]. Monocytes differentiate into multinucleated osteoclasts, thereby regulating osteoclastogenesis in bone metabolism [44]. RXRA, IL17RA, NFKB2, and TP53 are closely linked to osteoclastogenesis. RXRA plays a pivotal role in the vitamin D pathway, which participates in regulating osteoclastogenesis in the context of bone homeostasis [45]. The immunological and skeletal systems share numerous regulatory components, including the IL-17a receptor (IL17RA); the deletion of IL17RA reduces the number of osteoclast precursors and increases bone mass [46]. Osteoclastogenesis is negatively regulated by NFKB2 (p100); its blockade may prevent inflammation-mediated bone loss and bone deterioration in OP [47]. TP53 acts as a novel modulator of osteoblast-dependent osteoclastogenesis [48]. In this study, we identified two disulfidptosis clusters (clusters A and B) using the seven significant disulfidptosis modulators; we also identified two different disulfidptosis gene clusters (gene clusters A and B) based on 127 disulfidptosis-associated DEGs. Cluster B was verified to have a close association with monocyte immunity and elevated levels in gene expressions of RXRA, IL17RA, NFKB2, and TP53, indicating its association with osteoclastogenesis. Additionally, PCA was employed to calculate the disulfidptosis scores, thereby enabling the quantification of disulfidptosis signatures. Notably, cluster B and gene cluster B demonstrated higher disulfidptosis scores compared to cluster A and gene cluster A.

The RNA-seq analysis revealed significantly increased expression levels of the disulfidptosis genes OXSM and PRDX1 in OP patients in comparison to controls (Figure 10). These findings validate the involvement of these disulfidptosis modulators in OP and bring insights on their role in OP etiology, offering support for the hypothesis that disulfidptosis regulators play a vital role in OP progression. These disulfidptosis modulators may represent promising therapeutic targets for efforts to balance bone formation and resorption in OP. To date, this study firstly establishes a diagnostic cluster and an immunological landscape associated with disulfidptosis in OP. However, the in-depth mechanisms by which disulfidptosis regulators influence OP-related pathways and immune microenvironment require further experimental validation.

## CONCLUSIONS

In this study, we examined seven diagnostic disulfidptosis regulators and established a nomogram model with accurate prediction of OP incidence. By utilizing these vital disulfidptosis genes, two disulfidptosis signatures were identified, indicating that cluster B and gene cluster B may be more closely related to OP. To our knowledge, this research firstly elucidates a diagnostic cluster and an immunological landscape connected with disulfidptosis in OP.

## Supplementary Material

Supplementary Tables
