# Supplementary material for: RNA sequencing-based approaches to identifying disulfidptosis-related diagnostic clusters and immune landscapes in osteoporosis
Source: Aging (Albany NY). 2024 May 10;16(9):8198–216. doi: 10.18632/aging.205813 (PMC11131997; doi:10.18632/aging.205813)
Supplement: Supplementary Tables [file aging-16-205813-s001.pdf]

SUPPLEMENTARY TABLES

Supplementary Table 1. The detailed information of GO enrichment analysis in Figure 2J.

ONTOLOGY	ID	Description	p-value	p.adjust
BP	GO:0070527	platelet aggregation	1.04E-06	0.000432302
BP	GO:0034109	homotypic cell-cell adhesion	2.72E-06	0.000432302
BP	GO:0007596	blood coagulation	3.46E-06	0.000432302
BP	GO:0007599	hemostasis	3.66E-06	0.000432302
BP	GO:0050817	coagulation	3.71E-06	0.000432302
BP	GO:0051668	localization within membrane	1.67E-05	0.001535649
BP	GO:0030168	platelet activation	1.84E-05	0.001535649
BP	GO:0001894	tissue homeostasis	5.99E-05	0.004364239
BP	GO:0034329	cell junction assembly	7.16E-05	0.004635332
BP	GO:1900026	positive regulation of substrate adhesion-dependent cell spreading	7.98E-05	0.004649556
CC	GO:0098978	glutamatergic synapse	0.000182539	0.004467089
CC	GO:0005925	focal adhesion	0.000283152	0.004467089
CC	GO:0005924	cell-substrate adherens junction	0.000289373	0.004467089
CC	GO:0030055	cell-substrate junction	0.000297806	0.004467089
CC	GO:0042470	melanosome	0.00059076	0.005301481
CC	GO:0048770	pigment granule	0.00059076	0.005301481
CC	GO:0005884	actin filament	0.000647536	0.005301481
CC	GO:0030863	cortical cytoskeleton	0.000706864	0.005301481
CC	GO:0044448	cell cortex part	0.001763774	0.011758497
CC	GO:0036464	cytoplasmic ribonucleoprotein granule	0.002332005	0.013992032
MF	GO:0030957	Tat protein binding	0.003949442	0.043175984
MF	GO:0004312	fatty acid synthase activity	0.00434365	0.043175984
MF	GO:0031996	thioesterase binding	0.00434365	0.043175984
MF	GO:0099186	structural constituent of postsynapse	0.00434365	0.043175984
MF	GO:0005313	L-glutamate transmembrane transporter activity	0.005525471	0.043175984
MF	GO:0015172	acidic amino acid transmembrane transporter activity	0.005525471	0.043175984
MF	GO:0050998	nitric-oxide synthase binding	0.005525471	0.043175984
MF	GO:0017160	Ral GTPase binding	0.005919144	0.043175984
MF	GO:0072349	modified amino acid transmembrane transporter activity	0.005919144	0.043175984
MF	GO:0098918	structural constituent of synapse	0.00670609	0.043175984

Supplementary Table 2. The detailed information of KEGG enrichment analysis in Figure 2K.

Term	p-value	p.adjust
Focal adhesion	3.40E-05	0.001225119
Proteoglycans in cancer	3.60E-05	0.001225119
Salmonella infection	6.43E-05	0.00145771
Viral myocarditis	1.84E-04	0.003129081
Adherens junction	2.58E-04	0.003438715
Bacterial invasion of epithelial cells	3.03E-04	0.003438715
Leukocyte transendothelial migration	6.64E-04	0.006448343
Yersinia infection	9.56E-04	0.007437068
Fluid shear stress and atherosclerosis	9.84E-04	0.007437068
Phagosome	0.00117522	0.007991495
Tight junction	0.001449643	0.008961432
Neutrophil extracellular trap formation	0.001808145	0.010246156
Pathogenic Escherichia coli infection	0.001962254	0.010264096
Rap1 signaling pathway	0.002225638	0.01081024
Regulation of actin cytoskeleton	0.002395654	0.010860298
Shigellosis	0.00303791	0.012911117
MAPK signaling pathway	0.004307241	0.017228962
Fatty acid biosynthesis	0.006283878	0.023369719
Amyotrophic lateral sclerosis	0.006529775	0.023369719
Ferroptosis	0.014264049	0.048497766

Supplementary Table 3. The detailed information of GO enrichment analysis in Figure 6H.

ONTOLOGY	ID	Description	p-value	p.adjust
BP	GO:0019221	cytokine-mediated signaling pathway	4.02E-08	0.000115189
BP	GO:0030099	myeloid cell differentiation	1.13E-07	0.000162544
BP	GO:0032970	regulation of actin filament-based process	1.21E-06	0.00116027
BP	GO:0046824	positive regulation of nucleocytoplasmic transport	2.42E-06	0.001442425
BP	GO:0032956	regulation of actin cytoskeleton organization	2.52E-06	0.001442425
BP	GO:1903706	regulation of hemopoiesis	3.25E-06	0.001542839
CC	GO:0005925	focal adhesion	7.45E-09	1.22E-06
CC	GO:0030055	cell-substrate junction	9.42E-09	1.22E-06
CC	GO:0070820	tertiary granule	8.85E-06	0.000763914
CC	GO:0101002	ficolin-1-rich granule	2.12E-05	0.001374605
CC	GO:0034774	secretory granule lumen	0.000190749	0.007136597
CC	GO:0060205	cytoplasmic vesicle lumen	0.000204362	0.007136597
MF	GO:0061629	RNA polymerase II-specific DNA-binding transcription factor binding	4.26E-06	0.001141206
MF	GO:0003779	actin binding	6.29E-06	0.001141206
MF	GO:0016922	nuclear receptor binding	4.47E-05	0.003936513
MF	GO:0140297	DNA-binding transcription factor binding	5.51E-05	0.003936513
MF	GO:0019903	protein phosphatase binding	5.80E-05	0.003936513
MF	GO:0045296	cadherin binding	6.51E-05	0.003936513
